# Experiences and Attitudes Toward Telemedicine in an Adult Congenital Heart Disease Clinic: Lessons Learned from the COVID-19 Pandemic

**DOI:** 10.1007/s00246-024-03533-6

**Published:** 2024-06-05

**Authors:** Mia Shiue, Annique Nyman, Robert Karvell, Sara L. Partington, Tamar J. Preminger, Christian Reda, Emily Ruckdeschel, Kathleen Sullivan, Lynda Tobin, Sumeet S. Vaikunth, Joshua Saef, Bruke A. Tedla, Yuli Y. Kim

**Affiliations:** 1 Hospital of the University of Pennsylvania, 3400 Civic Center Boulevard, Philadelphia, PA 19104 USA; 2 Children’s Hospital of Philadelphia, 3401 Civic Center Boulevard, Philadelphia, PA 19104 USA; 3 Division of Cardiology, Hospital of the University of Pennsylvania, Perelman Center for Advanced Medicine, 2Nd Floor E. Pavilion, 3400 Civic Center Boulevard, Philadelphia, PA 19104 USA

**Keywords:** Telemedicine, COVID-19, Adult congenital heart disease, Patient experience, Patient attitudes

## Abstract

The coronavirus disease 2019 (COVID-19) pandemic has driven a broader adoption of telemedicine (TM). We aim to describe adult congenital heart disease (ACHD) patient experiences with TM and explore factors associated with positive attitude toward future TM visits. This is a cross-sectional, single-center study in an outpatient ACHD clinic from February to June, 2022. Between-group comparisons were made using Wilcoxon-Rank Sum, Chi-Square, or Fisher-Exact tests. Univariate logistic regression was performed for variables that could correlate with a “positive” attitude toward future TM visits. Significance was determined using an alpha level of 0.05. Of 262 patients (median age 33 years, 55% female, 81% White), 115 (44%) had a prior TM visit and 110 (96%) reported a positive experience. There were 64 (24%) with a positive attitude toward future TM visits. Concerns include lack of cardiac testing and limited quality of visit. Patients with visits every 3–6 months (Odds Ratio [OR] 2.44; *p* < 0.01) and prior TM visit (OR 1.89; *p* = 0.03) had higher odds of a positive attitude toward future TM, whereas males had lower odds (OR 0.53; *p* = 0.04). Age, annual income, disease complexity, distance from clinic, and employment status were not associated. There is high rate of satisfaction with TM among ACHD patients but only one-quarter indicated interest in using TM in the future. Factors associated with interest in TM visits are identified, and together with patient feedback, can be used to understand potential role of TM for the ACHD population in the post-pandemic era.

**Supplementary Information:**

The online version contains supplementary material available at 10.1007/s00246-024-03533-6.

## Introduction

The coronavirus disease 2019 (COVID-19) pandemic globally disrupted routine outpatient clinical care in unprecedented ways, including the broader adoption of telemedicine (TM) [1]. Adults with congenital heart disease are a growing population of young patients who utilize outpatient resources at a disproportionate rate compared to their peers [2], likely reflecting the complex needs of this chronic condition. Though TM has been implemented in adult congenital heart disease (ACHD) clinics previously [3, 4], the global experience of the COVID-19 pandemic provides an opportunity to examine ACHD patients’ experiences toward TM to better understand its potential role in longitudinal outpatient care moving forward.

We hypothesized that as a relatively younger population, ACHD patients would enjoy TM visits and may want to use TM with their ACHD provider in the future. The main objectives of this study are as follows:

1. To compare clinical and demographic characteristics of those who have had TM visits to those who have not.
2. To describe ACHD patients’ experiences of TM clinic visits during the COVID-19 pandemic.
3. To explore factors associated with a positive attitude toward having future TM visits.

## Methods

### Study Design

This is a cross-sectional survey of patients followed in an accredited North American ACHD program between February and June 2022. The inclusion criteria were patients ages 18 years or older with a history of congenital heart disease as well as those with congenital cardiomyopathy and heritable connective tissue disorders. Those with acquired heart conditions and new patients to the program at the time of recruitment were excluded. This paper questionnaire was administered in English. Patients were asked if someone (family member, care provider, etc.) would be helping them complete the survey and how that person would be helping them. Options of assistance include “read the questions to me,” “write down the answers I give,” “answer the questions for me,” “translate the questions into my language,” and “help in some other way.”

The questionnaire administered to patients can be found in table s1. Those who had a previous TM visit with their ACHD provider were asked questions about their experience. Specifically, “How would you rate your past telemedicine experience(s) during the COVID-19 pandemic?” was answered on a five-point Likert scale (Poor, Fair, Good, Very Good, Excellent). Responses of “Good,” “Very Good,” and “Excellent” were considered to have had a positive TM experience, while “Poor” and “Fair” corresponded to a negative TM experience.

All patients were queried about their concerns about TM visits during the pandemic and how to address those concerns. Patients were asked, “Do you currently have concerns about seeing your cardiologist for a telemedicine visit during the pandemic?” Those who responded “Yes” were asked to specify “What are some of your concerns about seeing your ACHD cardiologist for a telemedicine visit during the pandemic?” by selecting all that applied from a suggested list of concerns. This was followed by “What can your cardiology team do to help address your concerns about telemedicine visits during the pandemic?” with select all that apply responses or space for elaboration.

As a corollary to questions about TM visit concerns, patients were asked about any concerns regarding in-person office visits. Patients were asked, “Do you currently have concerns about seeing your cardiologist for a routine in-person visit during the pandemic?” Those who responded “Yes” were prompted to answer “What are some of your concerns about seeing your ACHD cardiologist for a routine in-person visit during the pandemic?” by selecting all that applied from a suggested list of concerns. This was followed by “What can your cardiology team do to help address your concerns about coming to a routine in-person visit during the pandemic?” with select all that apply responses or space for elaboration.

The outcome variable examining patient attitudes about pursuing TM in their future ACHD care was determined by the answer to: “For your next routine visit, would you prefer a telemedicine visit over an in-person visit with your ACHD cardiologist? (If the COVID-19 pandemic is still ongoing)” with those answering “yes” as having a positive attitude toward TM compared to this those who answered “maybe” (neutral attitude) or “no” (negative attitude).

To assess the profile of ACHD patients who may have a preference for their next visit to be remote, we queried various sociodemographic factors in the survey. Predictor variables include gender, marital status, race, ethnicity, state of residence, travel time to ACHD clinic, education level, employment status, annual income, health insurance, and presence of dependents in the household.

We sought to quantify the effects of fear specific to the pandemic as a potential driver of attitudes toward TM which was assessed by the *Fear of COVID-19 Scale* (FCV-19S) [5], a seven-item measure each on a 5-point scale with proven reliability and psychometric validity. FCV-19S was developed to access fear of COVID-19 among individuals and assist healthcare providers in designing appropriate programs that would address the fear. Scores range from 7 to 35, with a score of 7 to 22 considered “low fear” and 23 to 35 considered “high fear” of COVID-19.

Clinical data including congenital heart disease severity (Simple, Moderate, Complex) [6] were collected through retrospective chart review in the electronic health record. Survey and clinical data were collected and entered into REDCap [7].

### Statistical Methods

Categorical variables are presented as count (percentage) and continuous variables are presented as median and interquartile range (IQR). Patients were stratified according to their history of prior TM visit with their ACHD provider. Between-group comparisons were made using Wilcoxon-Rank Sum, Chi-Square, or Fisher-Exact testing. Patients were subsequently categorized according to their attitudes toward TM for future visits (positive, neutral, or negative). Univariate logistic regression was performed for variables of interest that could correlate with a “positive” response, using a P-value of 0.05. All statistical analyses were run in R (version 4.04) and RStudio. Significance was determined using an alpha level of 0.05.

## Results

There were 262 patients who comprised the study cohort (median age 33 [interquartile range (IQR) 27, 41] years, 55% female, 81% White). Table 1 describes the cohort sampled. This study population had 91 (36%) patients with complex CHD severity, and 169 patients (69%) have scheduled visits to the clinic at least annually (annually or every 2 years). Of those surveyed, 145 (55%) had a college or graduate degree. The median household income in Philadelphia in 2021 was about $50,000 [8], and 127 patients (51%) reported annual income to be above the median. There were 12 patients (5%) who had a high fear of COVID-19 during this phase of the pandemic, while 250 patients (95%) had a low fear of COVID-19.

**Table 1:** Clinical, Demographic, and Socioeconomic Characteristics of the Study Population

	*N* = 262
Age (Median [IQR])	33 [27, 41]
Gender (%)	
Female	145 (55.3)
Male	112 (42.7)
Non-Binary	4 (1.5)
Race (%)	
White	211 (80.5)
Black	25 (9.5)
Asian	12 (4.6)
Multiracial or other	14 (5.3)
Hispanic or latino (%)	14 (5.3)
ACHD severity (%)	
Simple	30 (12.0)
Moderate	129 (51.6)
Complex	91 (36.4)
Marital status (%)	
Single, never married	127 (48.7)
Married/remarried	108 (41.4)
Living with a partner	13 (5.0)
Separated/divorced	12 (4.6)
Widowed	1 (0.4)
Geographic area (%)	
Suburban area	178 (69.5)
Urban area	48 (18.8)
Rural area	30 (11.7)
Travel time to clinic (%)	
< 1 h	140 (53.4)
≥ 1 h	122 (46.6)
Education level (%)	
Less than high school	4 (1.5)
High school/GED	53 (20.2)
Vocational/technical diploma	14 (5.3)
Some college/university	28 (10.7)
Associate’s degree	16 (6.1)
College/university degree	82 (31.3)
Graduate/professional degree	63 (24.0)
Employment (%)	
Working full-time	161 (61.5)
Working part-time	39 (14.9)
Not currently working	30 (11.5)
Unable to work due to illness or disability	13 (5.0)
Student	8 (3.1)
Retired	6 (2.3)
Other	3 (1.1)
Caring for home or family	1 (0.4)
Have children under the age of 18 (%)	75 (28.6)
Self-reported annual income (%)	
< $20 K	57 (21.8)
Between $20-50 K	65 (24.8)
Between $50-100 K	81 (30.9)
Between $100-150 K	26 (9.9)
> $150 K	20 (7.6)
Insurance (%)	
Employer based	179 (68.3)
Insurance directly purchased	23 (8.8)
Medicare	24 (9.2)
Medicaid	43 (16.4)
TRICARE	3 (1.1)
Other	6 (2.3)
Frequency of cardiology visits (%)	
At least annually	169 (69.0)
Every 3 or 6 months	76 (31.0)

*ACHD* adult congenital heart disease, *IQR* interquartile range

### Characteristics of ACHD Patients with Prior TM Visits

Table 2 compares patients who had a prior TM visit to those who did not. Of the 262 subjects, 115 (44%) had at least one prior TM visit with their ACHD provider. There was no difference between those who had a prior TM visit compared to those who had not with respect to age, race, ethnicity, or marital status but patients who had a TM visit were more often female (*p* = 0.019) and had complex disease (*p* = 0.015). Travel time to clinic and geographical community did not differ nor did education level, employment status, or type of health insurance. The prior TM visit group had a higher proportion of those who earned less than $50,000 annually (*p* = 0.001).

**Table 2:** Patient Characteristics by History of Prior Telemedicine Visit

	Overall *N* = 262	No prior TM visit *N* = 147	Prior TM visit *N* = 115	*P*-value
Age (Median [IQR])	33 [27, 41]	33 [27, 44]	34 [27, 40]	0.747
Gender (%)				**0.026**
Female	145 (55.3)	73 (49.7)	72 (62.6)	
Male	112 (42.7)	73 (49.7)	39 (33.9)	
Non-binary	4 ( 1.5)	1 ( 0.7)	3 ( 2.6)	
ACHD severity (%)				**0.015**
Simple	30 (12.0)	19 (13.6)	11 (10.0)	
Moderate	129 (51.6)	81 (57.9)	48 (43.6)	
Complex	91 (36.4)	40 (28.6)	51 (46.4)	
Geographic area (%)				0.774
Rural area	30 (11.7)	15 (10.6)	15 (13.2)	
Suburban area	178 (69.5)	101 (71.1)	77 (67.5)	
Urban area	48 (18.8)	26 (18.3)	22 (19.3)	
Travel time to clinic (%)				0.138
< 1 h	140 (53.4)	85 (57.8)	55 (47.8)	
≥ 1 h	122 (46.6)	62 (42.2)	60 (52.2)	
Education level (%)				0.226
College Degree (Associate’s degree, College/University degree, graduate/professional degree)	161 (61.9)	95 (65.5)	66 (57.4)	
No College Degree (less than high school, high school/GED, vocational/technical diploma, some college/university)	99 (38.1)	50 (34.5)	49 (42.6)	
Employment (%)				0.115
Full time	161 (61.5)	97 (66.0)	64 (55.7)	
Not full time	101 (38.5)	50 (34.0)	51 (44.3)	
Have children under the age of 18 (%)	75 (28.6)	45 (30.6)	30 (26.1)	0.421
Income (%)				**0.001**
< $50 K	122 (49.0)	53 (38.7)	69 (61.6)	
≥ $50 K	127 (51.0)	84 (61.3)	43 (38.4)	
Insurance (%)				0.570
Private Insurance (Employer based, insurance directly purchased, other)	197 (75.2)	113 (76.9)	84 (73.0)	
Public Insurance (Medicare, Medicaid, TRICARE, government-assistance)	65 (24.8)	34 (23.1)	31 (27.0)	
Frequency of cardiology visits (%)				0.259
At least annually	169 (69.0)	97 (72.4)	72 (64.9)	
Every 3 or 6 months	76 (31.0)	37 (27.6)	39 (35.1)	
Fear of COVID-19 (%)				0.45
High	12 (4.6)	8 (5.4)	4 (3.5)	
Low	250 (95.4)	139 (94.6)	111 (96.5)	

*ACHD* adult congenital heart disease, *COVID-19* coronavirus disease 2019, *IQR* interquartile range, *TM* telemedicine

*P*-values < 0.05 are noted in bold.

### Experiences and Concerns about TM

Of the 115 patients who had a prior TM visit during the pandemic with their ACHD provider, 110 (96%) reported an overall positive experience using TM. Supportive examples include comments such as:

- “If no tests are needed it is an easier way to talk to your doctor. Doctor can also see the environment you live in.”
- “Don’t have to take off work or drive an hour to get to the appointment.”
- “For testing that takes place in different locations, I wouldn't mind telemedicine because I'd be traveling less.”
- “[TM] is SO much more convenient for routine care.”

There were 5 patients who did not have a positive experience using TM, of whom 2 did not want to pursue future TM visits with their ACHD provider. One commented, “I miss the personal aspect/chatting/catchup with the in-person visit.”

Table 3 presents patient concerns about TM visit, along with concerns about seeing their ACHD provider for an in-person visit. The greatest concerns about TM were about the “cardiologist not having enough information to treat them (i.e., no physical exam or testing, such as EKG or Echo)” in 40 (15%) patients and the “limited quality of a TM visit compared to in-person” in 33 (13%) patients. These sentiments were more prevalent in those who did *not* have a prior TM visit compared to those who did (*p* < 0.01). Comments included the following:

- “If I wasn’t due for a test, telemedicine would be fine.”
- “I don’t think I will get an accurate visit with a doctor…in person is much better”
- TM “still leaves Echo unaccounted for”
- “If I were to do [TM], I’d want to know important diagnosis isn’t being compromised.”

**Table 3:** Patient’s concerns and ways to help address concerns about telemedicine and in-person visits

	**Overall** ***N* = 262**
Concerns about seeing provider for a TM visit	
Cardiologist does not have enough information to treat me (no physical exam for testing, such as EKG or Echo)	40 (15.3)
Limited quality of visit (not the same as face-to-face)	33 (12.6)
Insurance coverage of telemedicine	8 (3.1)
Unstable Internet	6 (2.3)
No access to a working device	4 (1.5)
Poor audio/camera quality	4 (1.5)
Too technologically complicated	3 (1.2)
Breach of privacy	0 (0.0)
Other	5 (1.9)
How to help address concerns about TM visits	
Incorporate more remote digital test (Example: smartwatch or wearable watch for EKG testing)	36 (13.7)
Provide better technical support	5 (1.9)
Provide information on what the hospital is doing to protect my personal information	4 (1.5)
Other	15 (5.7)
Concerns about seeing provider for an in-person visit	
Time needed to take off	16 (6.1)
Transportation issues	16 (6.1)
Exposure to or contracting COVID-19	12 (4.6)
Inability to bring a family member along with me to my visit	8 (3.1)
Psychological distress	4 (1.5)
Caregiving responsibilities	3 (1.2)
Other	3 (1.2)
How to help address concern about in-person visits	
Open a more convenient clinic location	9 (3.4)
Have more flexible scheduling	7 (2.7)
Make parking more convenient and/or cheaper	5 (1.9)
Communicate and implement better COVID-19 safety policies	3 (1.2)
Provide access to a social worker or case manager	2 (0.8)
Provide on-site childcare	0 (0.0)
Other	7 (2.7)

*COVID-19* coronavirus disease 2019, *TM* telemedicine

The most favored way to help address concerns about TM visits, selected by 36 (14%) patients, was to “incorporate more remote digital test[s] (Example: smartwatch or wearable watch for EKG testing).”

There were 34 patients with concerns about seeing their provider for an in-person visit during the pandemic. The greatest concerns about in-person visits were about the need to take time off work and transportation issues in 16 (6%) patients. More patients with a prior TM visit were concerned about exposure to or contracting COVID-19 for an in-person visit, compared to those without a prior TM visit (*p* = 0.005). One patient hoped that the hospital would “continue mask wearing and continue to offer telemed[icine].” Another patient who had prior TM visits explained that “it is physically difficult for me to wear a mask due to pulmonary issues” and “because I am in the hospital, I have to wear one for long periods of time and my oxygen depletes.”

### Factors Associated with a Positive Attitude Toward Future TM Visits

Of the total cohort, 64 (24%), 119 (45%), and 76 (29%) patients had positive, neutral, and negative attitudes toward future TM visits with their ACHD provider, respectively. Table 4 describes variables tested for association with positive attitude. Patients with cardiology visits every 3 or 6 months (Odds Ratio [OR] 2.44, 95% Confidence Interval [CI] 1.33 – 4.48; *p* < 0.01), high fear of COVID-19 (OR 4.11, 95% CI 1.15–15.31; *p* = 0.03), and those with prior TM visit (OR 1.89, 95% CI 1.07–3.36; *p* = 0.03) had a higher odds of positive attitude toward future TM use. Male patients (OR 0.53, 95% CI 0.28–0.96; *p* = 0.04) had a lower odds of positive attitude toward future TM use compared to women. Age, race, disease complexity, distance from clinic, employment status, and annual income were not associated.

**Table 4:** Univariate analyses of factors associated with positive attitude toward TM

	**Odds ratio**	**95% confidence interval**	***P*-value**
Age	1.01	0.99–1.03	0.37
Gender			
Female	–	–	**–**
Male	0.53	0.28–0.96	**0.04**
Non-Binary	0.8	0.04–6.46	0.85
Race			
White	–	–	–
Black	1.1	0.38–2.81	0.85
Asian	1.04	0.22–3.64	0.96
Multiracial or other	1.25	0.33–3.91	0.72
ACHD complexity			
Simple	–	–	–
Moderate	2.21	0.79–7.92	0.17
Complex	2.46	0.85–8.96	0.12
Clinic drive			
< 1 hour	–	–	–
> 1 hour	1.66	0.94–2.96	0.08
Employment			
Not full time	–	–	–
Full time	0.89	0.50–1.60	0.7
Income			
< $50 K	–	–	–
> $50 K	0.68	0.38–1.22	0.2
Frequency of cardiology visit			
At least annually	–	–	**–**
Every 3 or 6 months	2.44	1.33–4.48	** < 0.01**
Fear of COVID-19			
Low	–	–	–
High	4.11	1.15–15.31	**0.03**
Prior Diagnosis of COVID-19	0.86	0.46–1.58	0.64
Vaccination status			
No	–	–	–
Yes	0.68	0.27–1.85	0.42
Prior telemedicine visit	1.89	1.07–3.36	**0.03**
Someone else helped complete survey	0.52	0.15–1.42	0.24

*ACHD* adult congenital heart disease, *COVID-19* coronavirus disease 2019, *TM* telemedicine

*P*-values < 0.05 are noted in bold.

## Discussion

The current study examined ACHD patient experience with TM during a world-wide health crisis, a distinct circumstance that profoundly disrupted usual access to outpatient care. Though many of these disruptions have since eased, knowing patients’ experiences and attitudes toward TM can help clinicians caring for those with ACHD understand how to implement TM, if at all, in future practice. Our study found that 44% of the sample had least one prior TM visit with their ACHD provider at the time of the study. These patients were more often female, had complex congenital heart disease, and earned less than $50,000 annually. Nearly all (96%) reported a positive TM experience. Of the total sample, positive attitude toward future TM visits was associated with female sex, more frequent outpatient clinic visits, high fear of COVID-19, and having had a prior TM visit.

Similar to our findings, literature examining ACHD patient satisfaction with TM shows high patient satisfaction with their TM experience [9–12]. On a scale from 0 as the “worst” visit to 10 as the “best” visit, the median rating for virtual visits by ACHD patients seen in the Massachusetts General Hospital was a 10 [9]. Another study conducted early in the pandemic from March 2020 to June 2020 at the Ohio State University and Nationwide Children’s Hospital found that 98% of their ACHD patients were satisfied with their care through TM [11].

We hypothesized that younger age would be associated with a more positive attitude toward future TM visits, but this was not found. Female sex, however, was a demographic that was not only associated with a positive attitude toward future TM visits but was also more preponderant in those who had had prior TM visits. A study in Australia examining patient satisfaction with TM during the COVID-19 pandemic found that being male was associated with a worse TM experience [13]. Why female patients would have better TM experiences is not clear, but increased TM use in females during the pandemic may be related to sex differences in patient engagement in their health care [14, 15]. It is possible that a positive attitude toward future TM-based care by females could reflect the gendered role of childcare and therefore the appeal of home-based medical encounters. We attempted to test for this by examining the need to care for family members or employment status but neither of these variables were significantly associated.

Other factors that could impact attitudes toward TM visits were distance from the cardiology clinic, fear of COVID-19, and the need for frequent visits to the cardiologist. Of those, fear of COVID-19 and higher frequency of clinic visits were associated, the former of which could be explained by concerns of contracting an illness during the in-person visit and the latter by the inconvenience of travel; both of which were common concerns cited about in-person visits during the pandemic. We believe it is intuitive that those who had a prior TM visit had higher odds of positive attitude toward future TM visits, especially as satisfaction was high for those who saw their ACHD provider previously by TM.

Despite high satisfaction with TM in those who had such encounters with their ACHD providers, only 24% of the sample had a positive attitude toward future TM visits with their ACHD provider. Patient concerns about TM thematically included not having enough testing or information for the cardiologist to care for them effectively and limited quality of visit. These sentiments echoed findings from the Massachusetts General Hospital ACHD program whose patients liked the convenience and cost of TM, but in-person visits were still preferred for a personal connection with the provider and showing physical problems [9]. Similarly, when asked about their preference for TM or in-person clinic visits, 76% of patients sampled in the Ohio State University program preferred TM if testing was done separately, and 18% preferred in-person visits [11]. Indeed, as a structural heart disease population acclimated to a schedule of routine cardiac testing starting in childhood (e.g., electrocardiogram, echocardiogram, advanced imaging such as cardiac magnetic resonance imaging and computed tomography), it is not surprising that TM alone without cardiac diagnostic testing could be perceived as “inaccurate,” “lacking,” or of “limited quality” for clinical encounters. Patients voiced interest in remote digital testing, such as a smartwatch for EKG testing, and defining the role of such technologies in longitudinal congenital cardiac care is ongoing and may be a direction to explore for TM-based care [16, 17]. Based on the current limitations of remote testing, we believe it is important to assure patients that TM would not replace vital cardiac testing.

Patient experience is a key metric related to greater self-care, adherence to medication, and improved clinical outcomes [18–20]. Investigating concerns and perceptions of TM is necessary for devising a post-pandemic care delivery paradigm that is acceptable to the patient. This point should be an essential aspect of a broader TM roll-out to ensure patient “buy-in” and partnership. The results of this study and others suggest that there is indeed a role for TM in outpatient ACHD clinics given high satisfaction ratings, but that patients may be more comfortable with TM as an adjunct instead of a replacement for in-person visits. We propose, given these findings, that TM visits could be incorporated into the ACHD provider “toolbox” as an alternative to in-person visits for specific clinical situations and patients, but not as a replacement for routine visits with cardiac testing. Examples included the following: follow-up after hospital discharge, medication titration follow-up, stable patient surveillance for those who are seen in the clinic more frequently than annually, reviewing testing results, patient education, and shared decision-making conversations. The result of a study comparing barriers to ACHD care of patients from urban or rural regions found that rural patients were more likely to cite distance from the clinic and needing to go into the city as barriers to in-person clinic appointments [21]. Indeed, we consider TM as a valuable means to expand access to ACHD care [22, 23].

Furthermore, TM itself has been found to help with patient engagement with their ACHD care and decrease rates of patient disengagement [24]. The ACHD population is also a relatively young population where more than 90% of the patients have access to a digital device that can facilitate TM [18]. We believe that given high satisfaction with this modality, TM is a means for patients to remain engaged in care for their chronic condition.

### Study Limitations

The survey questionnaire was administered to patients who came to the clinic for an in-person visit. Therefore, the study may be biased toward patients who are more comfortable coming into the clinic for their ACHD visit. It is possible that those who elected to complete the survey are distinct in other ways. For example, we acknowledge that over half of the sample had a college degree or greater, consistent with previous studies sampling our clinic [25, 26]. For these reasons, our results as a single-center study are not necessarily generalizable to the ACHD population as a whole. Patients’ experiences and attitudes toward telemedicine may differ according to the types of telemedicine services offered by different centers. There may also be a bias of the pandemic impacting patients’ concerns or attitudes toward TM, which may not be as relevant as the world has adapted to a “new normal.”

## Conclusion

Among ACHD patients, we describe the characteristics of those who had prior TM visits with their ACHD provider and document a high rate of satisfaction with their experiences. Despite this, only ~ 1/4 indicated a definite interest in using TM in the future with their ACHD provider with a documented concern for the lack of vital testing in TM and decreased quality of the visit. Frequent cardiology visits, fear of COVID-19, and prior experience with TM were associated with increased odds of a positive attitude toward future TM use. By examining patient attitudes and experiences regarding TM, these results can be used to determine how TM could be utilized in post-pandemic ACHD care.

## Supplementary Information

Below is the link to the electronic supplementary material.

Supplementary file1 (DOCX 22 kb)

## Abbreviations

ACHD: Adult Congenital heart disease
TM: Telemedicine
COVID-19: Coronavirus Disease- 2019
FCV-19S: Fear of COVID-19 Scale
IQR: Interquartile range
OR: Odds ratio
CI: Confidence interval
