# Supplementary material for: Experiences and Attitudes Toward Telemedicine in an Adult Congenital Heart Disease Clinic: Lessons Learned from the COVID-19 Pandemic
Source: Pediatr Cardiol. 2024 Jun 5;46(5):1185–93. doi: 10.1007/s00246-024-03533-6 (PMC12021723; doi:10.1007/s00246-024-03533-6)
Supplement: Supplementary file 1 — Supplementary file1 (DOCX 22 kb) [file 246_2024_3533_MOESM1_ESM.docx]

**SUPPLEMENTARY MATERIAL**

Table S1. Survey Asking Patients About Their Experiences and Attitudes Towards Telemedicine and In-Person Clinic Visits During the COVID-19 Pandemic

| **Will someone (family member, care provider, etc.) be helping you complete this survey?** |
| --- |
| Yes |
| No |
| **If “Yes,” how will the person (family member, care provider, etc.) be helping you? Please select all that apply.** |
| Read the questions to me |
| Write down the answers I give |
| Answer the questions for me |
| Translate the questions into my language |
| Help in some other way |
| **QUESTIONS ABOUT YOU** |
| **How old are you?** |
| **What is your gender?** |
| Female |
| Male |
| Non-Binary |
| Prefer not to answer |
| **What is your marital status?**  Single, never married  Separated/divorced  Married/remarried  Living with a partner  Widowed |
| **What is your race?**  Caucasian  Black/African American  Asian  American Indian/Alaska Native  Native Hawaiian/Other Pacific Islander  Other  Unknown |
| **If “Other” or “Unknown,” please explain.** |
| **What is your ethnicity?**  Hispanic or Latino  Not Hispanic or Latino |
| **What state do you live in?** |
| **Do you live in a…**  Suburban area  Urban area  Rural area |
| **About how long does it take you to get to your Adult Congenital Heart Disease (ACHD) clinic?**  Less than 30 minutes  Between 30 minutes to 1 hour  Between 1 to 2 hours  More than 2 hours |
| **What is your highest level of education?**  Less than high school  High school/GED  Vocational/technical diploma  Some college/university  Associate’s degree  College/university degree  Graduate/professional degree |
| **What is your employment status?**  Not currently working  Working full-time  Working part-time  Caring for home or family  Unemployed and looking for work  Unable to work due to illness or disability  Retired  Student  Other |
| **If you chose “Other,” please explain.** |
| **Do you typically work outside of the house?**  Yes  No  Sometimes  Other |
| **If you chose “Other,” please explain.** |
| **What is your occupation?** |
| **What is your annual income?**  Less than $20,000  $20,000 to $34,999  $35,000 to $49,999  $50,000 to $74,999  $75,000 to $99,999  $100,000 to $149,999  $150,000 or more |
| **Are you currently covered by any of the following types of health insurance or health coverage plans? Please select all that apply.**  Insurance through a current or former employer or union (of yours or of another family member)  Insurance purchased directly from an insurance company (by you or another family member)  Medicare, for people 65 and older, or people with certain disabilities  Medicaid, Medical Assistance, or any kind of government-assistance plan for those with low incomes or a disability  TRICARE or other military health care  VA (enrolled for VA health care)  Indian Health Service  Other |
| **If “Other,” please explain.** |
| **QUESTIONS ABOUT YOUR FAMILY** |
| **Do you have children?**  Yes  No |
| **How many children do you have?** |
| **How many of your children are under the age of 12?** |
| **How many of your children are between the ages of 12 and 17?** |
| **How many of your children are 18 or older?** |
| **Do you care for other family members in your house?**  Yes  No |
| **If so, who?** |
| **QUESTIONS ABOUT YOUR ACHD VISIT EXPERIENCES** |
| **On average, how often do you see your (or a) cardiologist?**  Every 3 months  Every 6 months  Annually  Every 2 years  Other |
| **If you chose “Other,” please explain** |
| **How would you rate your past IN-PERSON visit experiences during the COVID-19 pandemic?**  Poor  Fair  Good  Very Good  Excellent |
| **Do you currently have concerns about seeing your cardiologist for a routine IN-PERSON visit during the pandemic?**  Yes  No |
| **What are some of your concerns about seeing your ACHD cardiologist for a routine IN-PERSON visit during the pandemic? Select all that apply.**  Exposure to or contracting COVID-19  Caregiving responsibilities  Time needed to take off  Transportation issues  Inability to bring a family member along with me to my visit  Psychological distress  Other |
| **If you chose “Other,” please explain.** |
| **What can your cardiology team do to help address your concerns about coming to a routine IN-PERSON visit during the pandemic?**  Provide access to a social worker or case manager  Open a more convenient clinic location  Provide on-site childcare  Have more flexible scheduling  Communicate and implement better COVID-19 safety policies  Make parking more convenient and/or cheaper  Other |
| **If you chose “Other,” please explain.** |
| **QUESTIONS ABOUT YOUR ATTITUDE TOWARDS TELEMEDICINE** |
| **Do you currently have concerns about seeing your cardiologist for a TELEMEDICINE visit during the pandemic?**  Yes  No |
| **What are some of your concerns about seeing your ACHD cardiologist for a telemedicine visit during the pandemic? Select all that apply.**  No access to a working device  Unstable Internet  Poor audio/camera quality  Cardiologist does not have enough information to treat me (no physical exam for testing, such as EKG or Echo)  Limited quality of visit (not the same as face-to-face)  Breach of Privacy  Too technologically complicated  Insurance coverage of telemedicine  Other |
| **If you chose “Other,” please explain.** |
| **What can your cardiology team do to help address your concerns about TELEMEDICINE visits during the pandemic?**  Provide better technical support  Provide information on what the hospital is doing to protect my personal information  Incorporate more remote digital test (Example: smartwatch or wearable watch for EKG testing)  Other |
| **If you chose “Other,” please explain.** |
| **For your next routine visit, would you prefer a TELEMEDICINE visit over an IN-PERSON visit with your ACHD cardiologist? (If the COVID-19 Pandemic is still ongoing)**  Yes  No  Maybe |
| **Please explain.** |
| **After COVID-19, would you be interested to do some of your ACHD cardiology visits via TELEMEDICNE?**  Yes  No  Maybe |
| **Please explain.** |
| **PLEASE INDICATE YOUR LEVEL OF AGREEMENT WITH THE FOLLOWING STATEMENTS.** |
| **I am most afraid of coronavirus.**  Strongly Disagree  Disagree  Neither agree nor disagree  Agree  Strongly Agree |
| **It makes me uncomfortable to think about coronavirus.**  Strongly Disagree  Disagree  Neither agree nor disagree  Agree  Strongly Agree |
| **My hands become clammy when I think about coronavirus.**  Strongly Disagree  Disagree  Neither agree nor disagree  Agree  Strongly Agree |
| **I am afraid of losing my life because of coronavirus.**  Strongly Disagree  Disagree  Neither agree nor disagree  Agree  Strongly Agree |
| **When I watch news and stories about coronavirus on social media, I become nervous or anxious.**  Strongly Disagree  Disagree  Neither agree nor disagree  Agree  Strongly Agree |
| **I cannot sleep because I’m worried about getting coronavirus.**  Strongly Disagree  Disagree  Neither agree nor disagree  Agree  Strongly Agree |
| **My heart races or palpitates when I think about getting coronavirus.**  Strongly Disagree  Disagree  Neither agree nor disagree  Agree  Strongly Agree |
| *FOR PATIENTS WITH PRIOR ACHD TELEMEDICINE VISIT EXPERIENCE* |
| **How would you rate your past TELEMEDICINE experience(s) during the COVID-19 pandemic?**  Poor  Fair  Good  Very Good  Excellent |

ACHD, Adult Congenital Heart Disease; COVID-19, Coronavirus Disease 19.
